# Myositis-Related Interstitial Lung Disease: A Respiratory Physician’s Point of View

**DOI:** 10.3390/medicina57060599

**Published:** 2021-06-10

**Authors:** Yuko Waseda

**Affiliations:** Third Department of Internal Medicine, Faculty of Medical Sciences, University of Fukui, 23-3 Matsuoka Shimoaizuki, Eiheiji, Fukui 910-1193, Japan; yuwaseda@gmail.com; Tel.: +81-(776)-61-8355; Fax: +81-(776)-61-8111

**Keywords:** polymyositis, dermatomyositis, anti-ARS antibody, anti-MDA5 antibody, rapid progressive interstitial lung disease, progressive fibrosing interstitial lung disease

## Abstract

Idiopathic inflammatory myositis (IIM) is an umbrella term for diseases of unknown origin that cause muscle inflammation. Dermatomyositis and polymyositis are IIMs that commonly cause interstitial lung disease (ILD). When a patient presents with ILD, the evaluation of whether the case displays the characteristics of myositis should be determined by interview, physical examination, imaging findings, the measurement of myositis-related antibodies, and the determination of disease severity after diagnosis. Rapidly progressing anti-melanoma differentiation-associated gene 5 antibody-positive ILD may require rapid multi-drug therapy, while anti-aminoacyl tRNA synthetase (ARS) antibody-positive ILD can be treated with anti-inflammatory drugs. Importantly, however, anti-ARS antibody-positive ILD often recurs and sometimes develops into fibrosis. Early diagnosis is crucial for treatment, and we therefore need to clarify the features of myositis associated with ILD and suspect these pathologies early. This section reviews what clinicians need to look for and what findings are evaluated in patients when diagnosing myositis associated with ILD.

## 1. Introduction

Idiopathic inflammatory myositis (IIM) is an umbrella term for a spectrum of pathologies involving muscle inflammation of unknown origin, including dermatomyositis (DM), polymyositis (PM), sporadic inclusion body myositis, malignancy-associated myositis, and immune-mediated necrotizing myopathy. Among the IIMs, DM and PM are both connective tissue diseases (CTDs) that cause interstitial lung disease (ILD). PM can almost always be improved or prevented with anti-inflammatory drugs and DM is sometimes improved with anti-inflammatory drugs, but anti-melanoma differentiation-associated gene 5 (MDA5) antibody-positive ILD is sometimes unimproved by such agents and follows a rapidly progressive (RP) course.

Autoantibodies against aminoacyl-tRNA synthetases (ARSs) are detected in 25–35% of patients with IIM, and this condition is referred to as anti-synthetase syndrome (ASS). ASS represents a group of diseases strongly associated with arthritis, ILD, and so-called “mechanic’s hands” [1]. The combination of different classes of anti-inflammatory drugs, particularly steroids and immunosuppressive drugs, is effective in ASS, and these drugs thus represent the first line of therapy [2]. Early diagnosis is therefore important to enable prompt treatment. Although most cases respond readily to anti-inflammatory treatment, many relapse when pharmacotherapies are reduced or stopped. In some cases, fibrosis progresses to respiratory failure and the early administration of antifibrotic agents may be necessary. At present, however, patients with progressive fibrosis cannot be reliably identified at an early stage, so the evaluation of the progression of fibrosis within a relatively short period of time is necessary. In anti-MDA5 antibody-positive ILD, early diagnosis and early triple therapy with anti-inflammatory drugs are considered important [3], as about half of all patients with anti-MDA5 antibody-positive ILD die. However, some cases of anti-MDA5 antibody-positive ILD do not progress rapidly and do not necessarily require strong immunosuppression [4]. In any case, IIMs, particularly ASS and anti-MDA5 antibody-positive ILD, need to be treated early if treatment is actually required, and early diagnosis is therefore very important for clinicians.

Diagnostic criteria from the European League Against Rheumatism/American College of Rheumatology (EULAR/ACR) are shown in Table 1 [5]. The score using these criteria is characteristically higher if a muscle biopsy specimen is available for testing. Although the EULAR/ACR criteria do not mention the presence or absence of ILD, suspicion of IIM is important in patients with ILD because, as mentioned above, early treatment is crucial in ASS and anti-MDA5 antibody-positive ILD. The purpose of this review was to present the latest findings, with expert opinions, regarding what findings should be considered for suspected myositis-related ILD when examining ILD from the perspective of a respiratory physician. The paper is divided into an interview section, an objective findings section, and an examination section with reference to the EULAR/ACR classification to explain what is necessary in order to diagnose myositis-related ILD from the perspective of the respiratory physician.

## 2. Diagnostic Points

### 2.1. Interview

Questions regarding the characteristics of myositis include looking for the presence of progressive, symmetrical muscle weakness, particularly with a proximal muscle dominance. Specific questions for muscle weakness include: “Do you feel weakness in your thighs when climbing stairs?”, “Can you squat and stand up?”, “Do you feel weak when lifting a load onto an upper shelf?”, and “Do you find it difficult to support your neck?”

We need to check for the presence of any skin rashes, whether the fingers become pale and painful on exposure to cold air or water, and the presence of any dysphagia or esophageal motility disorders.

Other questions related to medication, hypersensitivity pneumonitis, pneumoconiosis, and familial factors should also be asked, and the possibility of these diseases should be investigated.

### 2.2. Physical Examination

#### 2.2.1. Inspection

**Figure 1:**
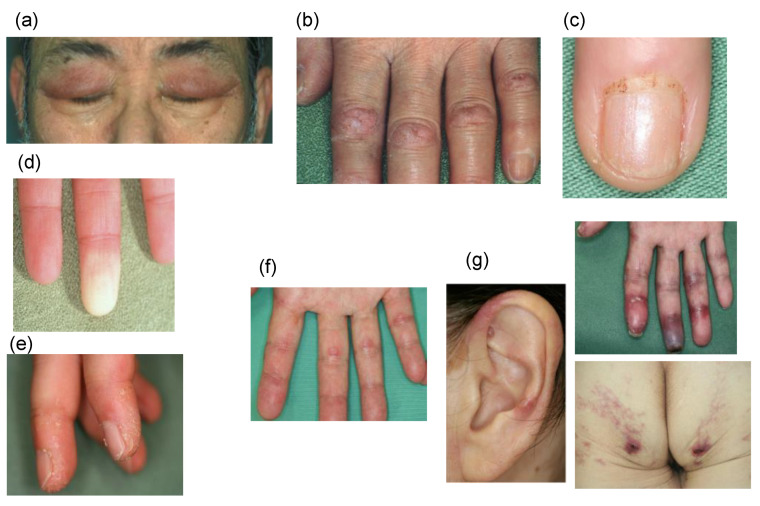
Skin manifestations of PM/DM. (**a**) Heliotrope rash. (**b**) Gottron’s sign. (**c**) Nail fold bleeding. (**d**) Raynaud’s phenomenon. (**e**) Anti-synthetase syndrome is often associated with mechanic’s hand. (**f**,**g**) Anti-MDA5 antibody-positive dermatomyositis is often associated with reverse Gottron’s sign (**f**), ulceration, and gangrene (**g**).

The performance of a basic visual examination is important because the diagnosis can be predicted to some extent by visual examination. In particular, sensory examination to determine whether the patient has exertional dyspnea is most important. Other findings characteristic of myositis include rashes of the face (e.g., heliotrope rash), hands (e.g., Gottron’s sign, mechanic’s hand, reverse Gottron’s sign, nail fold bleeding, periungual erythema, and Raynaud’s phenomenon), elbows, knees, and auricular skin (Figure 1). Skin rash in patients who are positive for anti-MDA5 antibody is particularly characterized by the inverted Gottron’s sign and vasculopathy (ulceration, purpura, or gangrene), which represent very important findings for early diagnosis.

#### 2.2.2. Auscultation

Most ILDs, including myositis-related ILDs, involve the dorsal aspect of the inferior lobes of the lungs more intensely than other areas of the lungs, and auscultation should always be performed not only anteriorly but also dorsally, as the inferior lobe usually contacts only the dorsal aspect, not the anterior chest. The presence of fine crackles and the timing of early, middle, and late crackles should be checked, along with the presence of coarse crackles, which reflect phlegmatic conditions. The absence of wheezes or rhonchi during forced expiration rules out airway disease.

### 2.3. Blood Tests

Routine screening tests for CTDs include antinuclear antibody (ANA) (fluorescent antibody method) with staining patterns, rheumatoid factor (RF), and anti-cytoplasmic antibodies such as anti-SS-A, myeloperoxidase-anti-neutrophil cytoplasmic antibodies (MPO-ANCA), and proteinase-3-anti-neutrophil cytoplasmic antibodies (PR3-ANCA). Myositis-specific autoantibodies include anti-ARS, anti-MDA5, anti-signal recognition particle (SRP), anti-Mi-2, anti-transcriptional intermediary factor (TIF)-1γ, anti-nuclear matrix protein (NXP)-2, anti-3-hydroxy-3-methylglutaryl-coenzyne A reductase (HMGCR), and anti-small ubiquitin-like modifier 1 activation enzyme (SAE) [6]. Among these, the anti-ARS and anti-MDA5 antibodies are often encountered in clinical practice, as they are frequently associated with ILD (Table 2). In addition, the serum concentrations of creatine kinase, aldolase, lactate dehydrogenase, aspartate aminotransferase, and alanine aminotransferase should be measured. Further, a serum ferritin level >1500 ng/mL is a poor prognostic factor in myositis-related ILDs, whereas a ferritin level >500 ng/mL is a poor prognostic factor in anti-MDA5 antibody-positive ILD [7].

Alveolar epithelium-derived biomarkers (Krebs von den Lungen (KL)-6, surfactant protein (SP)-D, SP-A) should be measured to evaluate the severity of ILD.

### 2.4. Imaging Test

**Figure 2:**
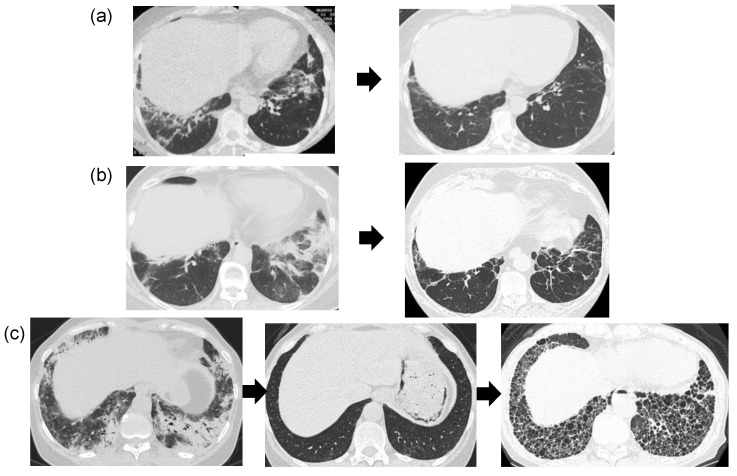
CT findings of anti-ARS antibody-positive ILD. (**a**) Prednisolone was administered, dense shadows along the bronchial vascular bundles improved, and prednisolone was discontinued after about 10 years. (**b**) Prednisolone and tacrolimus treatment left mild fibrosis after 4 years. (**c**) Prednisolone and cyclosporine A treatment achieved temporary improvement after 4 months, but fibrosis worsened after about 13 years.

Both anti-ARS and anti-MDA5 antibody-positive ILDs are considered to show certain characteristics in terms of imaging findings [8,9]. The early recognition of these characteristics and appropriate diagnosis and treatment may change the prognosis. High-resolution computed tomography of the chest is very important as the first screening in facilities where this modality is readily available. Anti-ARS antibody-positive ILDs can be classified into three groups: (1) those that improve during the course of treatment and show almost no abnormal shadowing; (2) those that improve, but then display mild fibrosis without progression; and (3) those that improve, but then exhibit clearly progressive fibrosis [10,11] (Figure 2). Almost all cases improve with anti-inflammatory drugs, and these drugs are therefore the first choice. In some cases, anti-fibrotic agents may be necessary for patients who later develop progressive fibrosis. However, the characteristics of patients who develop progressive fibrosis at the time of initial diagnosis are not yet clear, and will be the subject of further study.

### 2.5. Physiological Examination

In respiratory function tests, lung capacity (vital capacity: VC) and effortful lung capacity (forced VC: FVC) are used to evaluate ILD, but total lung capacity (TLC) and residual volume (RV) should also be measured as lung volume fractions upon initial examination or when the condition changes. RV may be increased in the presence of airway disease, and the possibility that fibrosis is not the only cause of decreased VC/FVC should be considered [12]. In addition, the lung diffusion capacity (DLco, DLco/VA) should be measured, but care should be taken to correct for the presence of anemia. If DLco is low and DLco/VA is also low compared to the decrease in VC, pulmonary hypertension (PH) should be considered [13].

Echocardiography is useful in screening for PH, and myositis-related ILD is less common, but can cause group 1 PH [14]; echocardiography should therefore be performed aggressively when hypoxemia on exertion is more severe than suggested from imaging.

### 2.6. Bronchoalveolar Lavage

Cytological evaluation of bronchoalveolar lavage fluid (BALF) in myositis-related ILD should include cell count and cell fractionation. Flow cytometry is mainly used to measure the CD4/8 ratio. As non-cytological evaluations, various cultures and PCR tests for *Pneumocystis jirovecii*, various viruses and acid-fast bacilli are performed [15]. In terms of cell fractionation, lymphocytes (>15%), neutrophils (>3%), and eosinophils (>1%) are generally considered to be elevated [16]. Although lymphocytes are often elevated in the BALF from individuals with myositis-related ILD, anti-inflammatory drugs may be effective even in the absence of lymphocyte elevation, and this is an auxiliary diagnosis for myositis-related ILD. The morphological characteristics of lymphocytes and other cells in BALF from cases of myositis-related ILD remain unclear.

### 2.7. Lung Biopsy

#### 2.7.1. Cryobiopsy

Transbronchial cryobiopsy (TBLC) is a new biopsy technique in which the tip of the cryoprobe is frozen, thus freezing the target tissue before collecting a sample. This technique can be performed using a flexible bronchoscope under sedation with spontaneous breathing. Although the specimens obtained are less than 1 cm in diameter and are inferior to those obtained by surgical lung biopsy (SLB), many allow better pathological diagnosis to be made than is possible in samples obtained by conventional transbronchial lung biopsy [17]. TBLC can be performed if a bronchoscope is available and offers the advantage of being able to be performed multiple times if necessary. Whether lung biopsy is actually useful for identifying myositis-related ILD remains unclear. Some patients show prolonged inflammation and the recurrence of ILD during steroid tapering, while others develop lung fibrosis resulting in end-stage ILD. In these latter cases, TBLC may be useful to assess the degree of inflammation and fibrosis in the lung [18]. However, among patients with ILD, particularly RP-ILD, bronchoscopy itself may cause exacerbation [19,20,21]. Care is therefore warranted.

#### 2.7.2. Surgical Lung Biopsy (SLB)

SLB is performed by a respiratory surgeon when necessary, since sufficient tissue can be obtained for pathological evaluation [22]. However, in reality, some facilities do not have a respiratory surgery department, and, except for a few facilities specializing in ILD, not all ILDs can be treated with SLB. Other problems include the inability to perform multiple specimen collections and the difficulty of performing SLB on elderly patients or patients with poor respiratory function. SLB is increasingly performed at a stage when TBLC is inadequate to reach an immediate diagnosis, but as with TBLC, lung histopathology is not always necessary and has limited indications.

### 2.8. Multidisciplinary Discussion (MDD)

As stated in the IPF guidelines for the American Thoracic Society (ATS)/European Respiratory Society (ERS)/Japanese Respiratory Society (JRS)/Latin American Thoracic Association (ALAT) and in the hypersensitivity pneumonitis (HP) guidelines for ATS/JRS/ALAT, the diagnosis of ILD can be improved by MDD among respiratory physicians, radiologists, and pathologists. The accuracy of ILD diagnosis is known to be enhanced by MDD among respiratory physicians, radiologists, and pathologists [23,24]. In addition, the inclusion of rheumatologists, dermatologists, nephrologists, and others in the discussion is important when CTD-ILD is suspected, because the appropriate diagnosis and treatment of ILD directly benefit patients [25,26]. However, relatively few facilities have all these specialists available for MDD. In the future, systems for online MDD and centralized MDD are highly desirable in order to reach an appropriate diagnosis at any hospital of any size in any region.

## 3. Characteristics of ILD by Antibody Profile

### 3.1. Anti-ARS Antibody

ARSs are enzymes responsible for the synthesis of aminoacyl-tRNAs, attaching amino acids to the 3′-terminal OH group of tRNAs that show an anticodon corresponding to each amino acid. Thus, 20 different anti-ARS antibodies would theoretically correspond to the number of amino acids, but the eight known anti-ARS antibodies are anti-Jo-1, anti-EJ, anti-PL-7, anti-PL-12, anti-KS, anti-OJ, anti-Zo, and anti-Ha. In myositis-related ILDs, anti-ARS antibody-positive patients show significantly better prognosis than those with negative antibodies and are more likely to recover with anti-inflammatory drugs, particularly when steroids and immunosuppressive drugs are administered simultaneously [27]. In terms of antibodies, the anti-Jo-1, anti-EJ, and anti-PL-7 antibodies are associated with relatively high incidences of classical myositis, whereas anti-KS and anti-OJ antibodies are associated with relatively high incidences of interstitial pneumonia without myositis. In addition, anti-Jo-1, anti-EJ, and anti-PL-7 antibodies may cause myositis over time, while anti-PL-12, anti-KS, and anti-OJ antibodies often cause only ILD [28]. Furthermore, anti-PL-7/PL-12 antibodies are associated with a worse prognosis than anti-Jo-1 antibodies [29], and anti-PL-7 antibodies represent a higher risk of relapse [30]. Thus, various characteristics can be seen in the types of ARS antibodies used. Anti-Jo-1 antibodies can be measured by double immunodiffusion, enzyme-linked immunosorbent assay (ELISA), and chemiluminescent immunoassay (CLEIA). However, the immunoprecipitation method is complicated and time-consuming [31]. Recently, the easy measurement of most anti-ARS antibodies has become possible using ELISA, but only anti-Jo-1, anti-EJ, anti-PL-7, anti-PL-12, and anti-KS antibodies can be measured, not anti-OJ antibodies [32]. EUROLINE Myositis profile 3 (Euroimmun, Lübeck, Germany), which measures anti-ARS antibodies using the Lineblot method [33], can measure only anti-Jo-1, anti-EJ, anti-PL-7, anti-PL-12, and anti-OJ antibodies. Anti-KS antibodies cannot be measured. In addition, the specificity of this assay is considered poor, and the false-positive and false-negative rates are relatively high [34,35].

### 3.2. Anti-MDA5 Antibody

MDA5 is a member of the RIG-I family of proteins and plays an important role in the innate immune system during viral infection [36]. Anti-MDA5 antibody is an autoantibody found specifically in DM. Initially, anti-MDA5 antibodies were found in cases of clinically amyopathic DM (CADM), a disease with a DM rash but no clinical evidence of myositis [37]. Subsequently, anti-MDA5 antibodies were found not only in CADM, but also in classical DM that fulfilled the classification criteria of Bohan and Peter [38]. Although the pathogenesis of autoimmune diseases involving anti-MDA5 antibodies has not been fully elucidated, viral infection is reportedly associated with the development of idiopathic inflammatory muscle diseases [39,40].

An important clinical feature of anti-MDA5 antibody-positive DM is its close association with RP-ILD. More than 50% of anti-MDA5-positive DM patients have been reported to develop RP-ILD in Japan [37,41]. On the other hand, in many foreign countries the frequency of RP-ILD in MDA5 is low, but some reports have described more than 50% of MDA5 patients as having RP-ILD, suggesting that RP-ILD is not a disease unique to Japan or Asia [42].

Regarding the relationship between anti-MDA5 antibody titer and prognosis, this titer was reportedly higher among non-survivors [43], the changes in titer during the course of treatment were greater in the survivor group than in the non-survivor group [44], and the titer of anti-MDA5 antibody was decreased when active disease changed to inactive disease [45]. The titer of anti-MDA5 antibody reflects the disease activity [46]. In terms of treatment, early triple therapy with prednisolone, intravenous cyclophosphamide, and tacrolimus may improve the prognosis of RP-ILD [3]. The early suspicion and treatment of anti-MDA5 antibody-positive ILD is therefore important. However, some cases show that chronic forms of ILD have better prognosis than acute and subacute forms, and others can be saved without triple therapy [47]. The chronic form may not require aggressive triple therapy [48]. Factors associated with early death include a low partial pressure of arterial oxygen (PaO_2_), a low percent-predicted FVC (%FVC), acute onset, high ferritin, and a high spread of consolidation [9,49].

## 4. Discussion

IIM is characterized by different types of antibodies, but the two most important antibodies for myositis-related ILDs are anti-ARS and anti-MDA5.

Comparing the prognosis of anti-ARS antibody- and anti-MDA5 antibody-positive ILDs, anti-ARS antibody-positive ILDs have a good prognosis, while anti-MDA5 antibody-positive ILDs show a significantly worse prognosis, and indications for treatment need to be determined as soon as possible [50].

In addition to the evaluation of antibodies, the priority is for the clinician to first suspect IIM by conducting a thorough interview when observing ILD and then examine the whole body, especially for Gottron’s sign, heliotrope rash, and mechanic’s hand.

In cases of RP-ILD, anti-MDA5 antibody-positive ILD should be suspected at that time and early triple therapy should be initiated. Sato et al. examined 497 patients with myositis-related ILDs in the JAMI (Japanese patients with myositis-associated ILD) study and found that poor prognostic factors in multivariate analysis included: (1) onset at 60 years or older; (2) anti-MDA5 antibody positivity; (3) C-reactive protein (CRP) > 1 mg/dL; and (4) SpO_2_ <95% [51]. In the JAMI cohort of 497 patients and 111 additional patients, Gono et al. found that the combination of CRP > 0.8 mg/dL and KL-6 >1000 U/mL was associated with an over 50% risk of mortality [52].

In addition, CD206, chitotriosidase, chitinase-3-like 1 protein (YKL-40), and CD163 have been reported as novel prognostic biomarkers, and the results of further studies are awaited [48,53,54,55].

**Figure 3:**
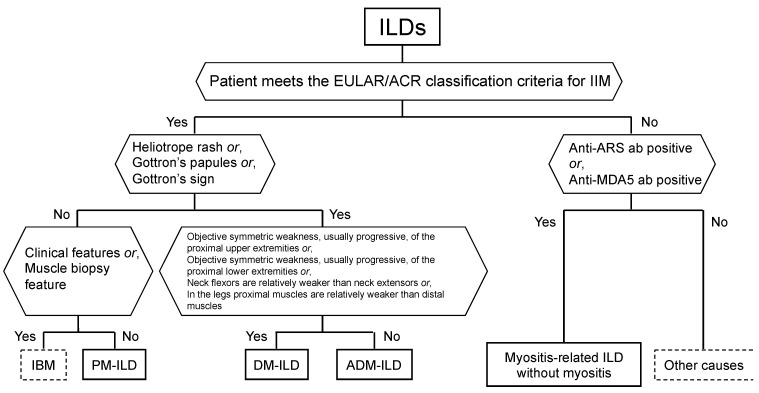
Classification tree for subgroups of adult myositis-related interstitial lung disease (ILD). A patient must first meet the EULAR/ACR classification criteria for inflammatory myopathies (IIM). The patient can then be sub-classified using the classification tree (modified from the figure in Reference [5]). ARS, aminoacyl-tRNA synthetase; ab, antibody; MDA5, melanoma differentiation-associated gene 5; IBM, inclusion body myositis; PM, polymyositis; DM, dermatomyositis; ADM, amyopathic dermatomyositis.

Figure 3 shows a revised version of the diagnostic flowchart described by Lundberg et al. [5]. We believe that the most important point is to suspect myositis-related ILDs and to develop the ability to diagnose myositis-related ILDs by combining interviews, physical examinations, and imaging findings. The suspicion of IIM is important, followed by the measurement of antibodies and the identification of factors associated with poor prognosis as soon as possible, then providing adequate treatment as soon as possible. In addition, even if the patient does not meet the criteria for IIM, clinicians should consider myositis-related ILD in those patients who show positive results for anti-ARS or anti-MDA5 antibodies.

## 5. Conclusions

The basis for identifying myositis ILD is an interview and physical examination. If anti-MDA5 antibody-positive ILD is suspected, prognostic factors should be evaluated as soon as possible, and, if necessary, multi-drug anti-inflammatory therapy should be administered as soon as possible. In addition, some patients with chronic anti-ARS antibody-positive ILD may develop progressive fibrosis, which requires a thorough evaluation for progression during the course of the disease.

## Figures and Tables

**Table 1:** Point scores for the European League Against Rheumatism/American College of Rheumatology classification criteria for adult and juvenile idiopathic inflammatory myopathies, to be used in the absence of better explanations for symptoms or signs (from the figure in Reference [5]).

	Points	
Variable	No Biopsy	Biopsy
Age at onset of first related symptoms		
18–40 years	1.3	1.5
≥40 years	2.1	2.2
Muscle weakness		
Objective symmetric weakness, usually progressive, of proximal upper extremities	0.7	0.7
Objective symmetric weakness, usually progressive, of proximal lower extremities	0.8	0.5
Neck flexors are relatively weaker than neck extensors	1.9	1.6
In the legs, proximal muscles are relatively weaker than distal muscles	0.9	1.2
Skin manifestations		
Heliotrope rash	3.1	3.2
Gottron’s papules	2.1	2.7
Gottron’s sign	3.3	3.7
Other clinical manifestations		
Dysphagia or esophageal dysmotility	0.7	0.6
Laboratory measurements		
Anti-Jo-1 (anti-histidyl-tRNA synthetase) autoantibody positivity	3.9	3.8
Elevated serum levels of creatine kinase (CK)★ or lactate dehydrogenase (LDH)★ or aspartate aminotransferase (ASAT/AST/SGOT)★ or alanine aminotransferase (ALAT/ALT/SGPT)★	1.3	1.4
Muscle biopsy features		
Endomysial infiltration of mononuclear cells surrounding, but not invading, myofibers		1.7
Perimysial and/or perivascular infiltration of mononuclear cells		1.2
Perifascicular atrophy		1.9
Rimmed vacuoles		3.1

★ Serum levels above upper limit of normal.

**Table 2:** Myositis-specific autoantibodies (taken with some modifications from Reference [6]).

Autoantibodies	Frequency	Significance
Anti-aminoacyl-tRNA synthetase (ARS)	~30%	Anti-synthetase syndrome: myositis, ILD, arthritis, Raynaud’s phenomenon, fever, mechanic’s hand
Anti-Jo-1	15–20%	
Anti-PL-7	<5%	
Anti-PL-12	<5%	
Anti-OJ	<5%	
Anti-EJ	<5%	
Anti-KS	<5%	
Anti-phenylalanyl-tRNA synthetase	<1%	
Anti-tyrosyl-tRNA synthetase	<1%	
Anti-SRP	5%	Severe disease, resistant to treatment, recurrent
Anti-Mi-2	5–10%	Childhood and adult DM
Anti-MDA5	20–35% of DM	CADM, rapidly progressive ILD
Anti-TIF1-γ	20% of DM	DM, malignancy-associated DM
Anti-NXP2 (MJ)	3–15%	DM, JDM, malignancy, skin calcification in children
Anti-HMGCR	5–8%	Necrotizing myopathy, statin-induced myopathy
Anti-SAE	2–8% of DM	DM

ARS: aminoacyl tRNA synthetase; ILD: interstitial lung disease; DM: dermatomyositis; MDA5: melanoma differentiation-associated gene 5; CADM: clinically amyopathic dermatomyositis; TIF-1γ: transcriptional intermediary factor-1 gamma; NXP2: nuclear matrix protein 2; JDM: juvenile dermatomyositis; SRP: signal recognition particle; HMGCR: 3-hydroxy-3-methylglutaryl-coenzyme A reductase; SAE: small ubiquitin-like modifier activating enzyme.

## Data Availability

Not applicable.
